# Optimizing Forest Aboveground Biomass Models with Multi-Parameter Integration

**DOI:** 10.3390/s26061974

**Published:** 2026-03-21

**Authors:** Xinyi Liu, Yang Zhao

**Affiliations:** 1 School of Computer Engineering, Chengdu Technological University, Chengdu 611730, China; 2 College of Geography and Planning, Chengdu University of Technology, Chengdu 610059, China; byteyang@foxmail.com

**Keywords:** forest AGB estimation, multi-parameter, machine learning, models

## Abstract

**Highlights:**

**What are the main findings?**

- Three forest AGB estimation models (univariate function, multivariate regression, decision tree) were developed. The optimized decision tree model achieved the highest accuracy (R^2^ = 0.8) without overestimation bias.
- Traditional univariate (power function: R^2^ = 0.7349) and multivariate regression (best R^2^ = 0.517) models showed insufficient precision with overestimation or underestimation.

**What are the implications of the main findings?**

- Multi-parameter integration combined with machine learning effectively captures non-linear relationships, providing a reliable paradigm for AGB estimation in heterogeneous landscapes.
- Incorporating ecological parameters (e.g., LAI, CIg) enhances estimation completeness, supporting regional carbon stock assessments and forest management under carbon neutrality goals.

**Abstract:**

Forests constitute a fundamental component of terrestrial carbon stocks and play a pivotal role in mitigating climate change through carbon sequestration. Accurate estimation of aboveground biomass (AGB) is essential for quantifying carbon budgets and informing ecosystem models. This study takes Wolong Nature Reserve in Sichuan Province, China, a mountainous area with high vegetation coverage and diverse forest types dominated by coniferous and mixed forests, as the study area, and constructs and evaluates AGB estimation models by integrating canopy height, leaf area index (LAI), vegetation indices (VIs), and topographic variables. Initially, univariate parametric models (linear, exponential, logarithmic, power, and polynomial) were established to relate canopy height to field-measured AGB. Subsequently, multivariate regression models incorporating VIs, LAI, and topographic metrics were developed. Finally, a decision tree-based machine learning framework was implemented to exploit the combined predictor set. Comparative analysis revealed that both canopy height-based and conventional multivariate regression models tended to overestimate AGB, limiting their applicability for large-scale assessments. In contrast, the optimized decision tree model, following parameter tuning and cross-validation, achieved superior predictive accuracy.

## 1. Introduction

Forest carbon sink represents a crucial strategy for the Chinese government to address climate change, exhibiting significant cost advantages compared to industrial emission reduction [1]. As the main body of terrestrial ecosystems, forests absorb CO_2_ and release O_2_ through photosynthesis, fixing CO_2_ in soil and plants, and storing two-thirds of the organic carbon in terrestrial ecosystems. Foreign scholars initiated research on forest carbon sink relatively early, beginning in the late 1960s, while domestic studies on forest carbon sink started in the late 1970s and currently remain in the initial stage [2]. The carbon sequestration capacity of forests can be evaluated by calculating forest carbon storage and carbon sink capacity. The primary calculation methods for forest carbon storage and carbon sink capacity include the biomass method, volume method, carbon density method, and carbon balance method [3], among which the biomass method is the most widely used.

Forest aboveground biomass (AGB) plays a critical role in assessing carbon sequestration capacity and serves as a key indicator of forest carbon stocks [4]. In the late 1970s, China began measuring tree aboveground biomass and developing biomass equations [5]. Subsequent research gradually expanded to cover most of China’s climatic zones and forest types [6]. Various forms of allometric models have been employed for AGB estimation [7,8], with AGB most commonly modeled as a power function of diameter at breast height (DBH). The integration of multi-sensor data, such as optical, radar, and LiDAR, allows for the fusion of different sensor characteristics, which can enhance AGB estimation models by incorporating structural attributes of the forest canopy [9,10]. Most existing studies have focused on generating large-scale AGB maps, often applying a uniform allometric model to estimate AGB for the same tree species. However, these approaches tend to overlook the spatial variability in AGB distribution under different research regions and topographic conditions, as well as the ecological significance of forest structural parameters in AGB estimation. In reality, the values of these parameters not only vary across forest types but also change within the same forest type depending on stand age, stand density, and site conditions [11,12,13]. Furthermore, nearly all allometric models are developed based on DBH or tree height, meaning that AGB estimates derived from such models strictly represent only stem biomass. In practice, total forest AGB consists of three components: stems, branches, and leaves. Consequently, allometric models that rely solely on tree height and DBH introduce inherent estimation errors.

Accurate estimation of forest AGB is of great significance for sustainable forest management and regional carbon stock assessment [14]. Forest aboveground biomass primarily consists of three components: stems, branches, and leaves. Within the tree layer, stems account for approximately 55.5–94.2% of AGB, branches for 2.9–26.0%, and leaves for 2.9–31.6% [15], indicating that stem biomass constitutes the dominant portion of forest AGB. Therefore, AGB models based on forest height can directly reflect stem biomass values. Although the contributions of branches and leaves to total AGB are relatively smaller, they remain integral components of the forest ecosystem. Forests absorb carbon dioxide and water, converting them into organic matter through photosynthesis, which subsequently accumulates as forest AGB. The canopy, composed of branches and leaves, plays a critical role in this process by intercepting and distributing incoming energy, thereby influencing AGB accumulation. As one of the fundamental vegetation parameters, the leaf area index (LAI) characterizes key biophysical processes in forests, including photosynthesis, transpiration, respiration, nutrient cycling, and precipitation interception. LAI is an essential variable in determining gas exchange within forests and is closely related to AGB accumulation and vegetation growth dynamics. Consequently, accurate estimation of branch and leaf biomass is of great importance for understanding overall forest AGB distribution [16,17].

When estimating forest AGB using multispectral remote sensing data, spectral features, texture information, and vegetation indices (VIs) derived from multispectral imagery are typically utilized to establish relationships with field-measured AGB [18,19,20,21]. In contrast, AGB estimation based on LiDAR or synthetic aperture radar (SAR) technology primarily relies on extracted tree height information to model AGB [22,23,24,25,26,27]. Additionally, other parameters correlated with ground-measured AGB include LAI and forest canopy closure [15,28]. In previous studies on forest AGB estimation, leaf biomass has often been overlooked.

The study area is the Wolong Nature Reserve in Sichuan Province, which features rich forest resources and diverse ecosystems. Current research on Wolong Nature Reserve encompasses diverse dimensions, including forest community analysis, landscape pattern assessment [29], and ecological environment evaluation. Specifically, studies have focused on ramet populations across different zones to analyze the size structure and formation mechanisms of forest gaps, as well as the structural characteristics of gap-generating trees [30,31,32]. Other researchers have investigated the impact of edaphic factors (e.g., soil properties) on spatial distribution patterns [33,34]. Additionally, comprehensive functional evaluations of the reserve have been conducted by examining factors influencing giant panda habitat quality [35,36], while studies on vegetation–geography relationships within the reserve have also been documented [37,38]. Notably, a conspicuous research gap persists: investigations explicitly targeting forest AGB estimation in this region remain disproportionately scarce [39].

To fill these gaps, this study integrates canopy height, LAI, multispectral vegetation indices, and topographic indices to analyze their correlations with field-measured AGB. Given that the dominant tree species in the region are larch (*Larix* spp.) and spruce (*Picea* spp.), with the remaining forested areas primarily consisting of mixed stands of shrubs and young trees, three distinct allometric models are employed to estimate AGB: one for larch, one for spruce, and one for mixed forests. While forest canopy height and LAI are generated from previously validated models with high accuracy, we construct three types of AGB estimation models: univariate function models, multivariate regression models, and decision tree-based machine learning models. The estimation accuracy and bias of different models are compared to identify a high-precision model suitable for mountain forest landscapes. This approach overcomes the limitation of traditional AGB models that rely only on diameter at breast height and tree height while ignoring branch and leaf biomass, thereby improving the accuracy and comprehensiveness of AGB estimation. The results can provide technical support and data reference for forest carbon stock assessment and forest management in Wolong Nature Reserve under the carbon neutrality goal.

## 2. Materials and Methods

### 2.1. Study Area

**Figure 1:**
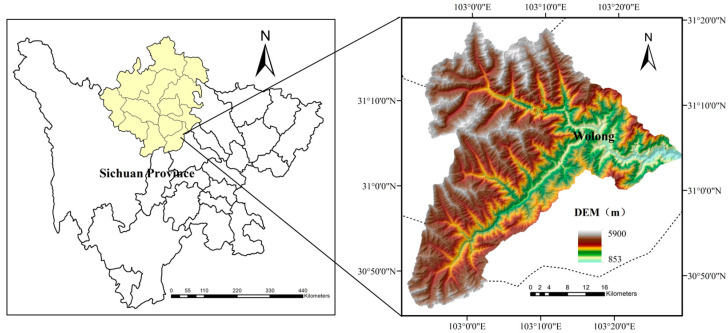
Location map of the Wolong Nature Reserve and its DEM [40].

The study area is the Wolong Nature Reserve in Wenchuan County, Sichuan Province, located between 102°51′44″ E–103°29′11″ E longitude and 30°45′37″ N–31°19′23″ N latitude, covering a total area of 1736.81 km^2^. As one of China’s earliest established comprehensive national nature reserves, Wolong is situated at the transition zone between mountainous and plain regions. It borders Siguniang Mountain to the west, with the highest elevation reaching 6132 m, and extends eastward to Yingxiu Town in Wenchuan City, where the lowest elevation is 898 m. The overall average elevation in the reserve is 3544 m (Figure 1).

The region exhibits significant topographic variation, with an average slope of 31.4°, and areas with slopes exceeding 25° accounting for 74.7% of the total area. Vegetation coverage within the reserve is generally high, with more than 60% of the area having a vegetation cover greater than 80% [40]. Figure 1 presents a location map of the study area and its Digital Elevation Model (DEM). The DEM data was obtained from ESA’s PALSAR 12.5 m resolution.

### 2.2. Data Acquisition and Preprocessing

#### 2.2.1. Sample Plot Survey Data

A field survey was conducted in October 2020, during which 64 sample plots were recorded. The plots were classified into two types, namely 10 × 10 m and 5 × 5 m, of which both square in shape, with elevations ranging from 940 m to 4193 m. Based on tree composition, the plots were categorized into three forest types: 15 broadleaf forest (BF) plots, 20 coniferous forest (CF) plots, and 29 mixed forest (MF) plots. The dominant tree species in the study area were larch (Larix gmelinii) and Chinese fir (Cunninghamia lanceolata).

The field measurement data included the geographic coordinates, elevation, slope, aspect, and canopy closure (CC) of each sample plot. Additionally, approximately 20 g of topsoil was collected from the four corners of each plot. For arboreal forests, the following measurements were recorded: diameter at breast height (DBH), root diameter, tree height (h), crown width, crown shape, and LAI. Healthy branches were sampled, and their fresh weight (branches and leaves) was measured. These samples were then sealed in plastic bags and transported to the laboratory, where they were oven-dried at 100 °C ± 2 °C before determining the dry weight of branches and leaves. For shrubs and small trees, measurements varied based on tree height. For trees taller than 1.3 m, DBH, root diameter, tree height, crown width, and LAI were recorded. For trees shorter than 1.3 m, basal diameter, root diameter, tree height, crown width, and LAI were measured. For shrubs, clump diameter, root diameter, crown diameter, height, and LAI were recorded. Similarly to arboreal forests, healthy branches were sampled for fresh weight measurement, sealed in plastic bags, and later oven-dried at 100 °C ± 2 °C to determine their dry weight. DBH was measured using a diameter tape, and LAI was obtained using the “LAISmart” device developed by Beijing Normal University [41,42]. During LAI measurements, values were collected from the four corners of each sample plot, and the effective LAI was calculated as the average of them. Finally, the Average Clumping Index was used to convert the effective LAI into the true LAI. Canopy closure for each sample plot was estimated using the upward-looking method.

Based on the allometric models for major tree species calculated by Tang et al. [14], the biomass of stems, branches, and leaves for each tree species was computed separately, with the total sum representing the biomass of the species. The calculations were performed using a power-law univariate equation, which has two forms: one using diameter at breast height (DBH, abbreviated as D) as the base and the other using the product of the square of DBH and tree height (D^2^H) as the base. The equations are expressed as follows in Equations (1) and (2).

(1) W=a∗DBH

(2) $$W=a*{\left(D^{2}H\right)}^{b}$$

In these equations, *W* represents biomass (kg), *DBH* is the measured diameter at breast height (cm), and *a* and *b* are coefficients. The coefficients for dominant tree species are listed in Table 1. Specifically, Equation (2) was used for *Larix gmelinii* and *Abies* spp., while Equation (1) was applied to *Picea* spp.

Zhang Y and Liang S proposed allometric growth models suitable for mixed forests in Sichuan Province when creating an AGB map of China [43,44]. Equations (3) and (4) were established based on univariate diameter at breast height (DBH) and two variables, DBH and tree height, respectively:

(3) $$W=0.2869*{DBH}^{2.0527}$$

(4) $$W=0.0573*{\left(D^{2}H\right)}^{0.8862}$$

The biomass of each individual tree in the sample plot was calculated using the corresponding species-specific biomass equation. The total AGB of the plot is the sum of the biomass values for each individual tree within the plot. The biomass per unit area is calculated by dividing the total AGB by the plot area, with the unit expressed as t/ha.

Table 2 presents the basic information of the 64 sample plots in the Wolong Nature Reserve. Among these, 20 plots are coniferous forests, 15 plots are broadleaf forests, and 29 plots are mixed forests. The tree height of the coniferous forests in the study area ranges from 8.90 to 22.20 m, with an average of 15.50 m and a standard deviation of 2.23 m. The range of tree diameter at breast height is 15.00 to 27.23 cm, with an average of 15.09 cm and a standard deviation of 3.30 cm. The east–west crown width ranges from 3.70 to 7.60 m, with an average of 5.70 m and a standard deviation of 0.91 m. The north–south crown width ranges from 2.10 to 8.80 m, with an average of 5.33 m and a standard deviation of 0.85 m. The leaf area index was measured using LAISmart ranges from 1.80 to 2.70, with an average of 2.33 and a standard deviation of 0.56. The measured canopy closure in the field ranges from 20.56% to 95.89%, with an average of 61.23% and a standard deviation of 13.75%. The unit area forest biomass in coniferous forests ranges from 1.17 to 194.23 t/ha, with an average of 86.23 t/ha and a standard deviation of 35.74 t/ha.

In the broadleaf forests of the study area, the tree height ranges from 2.30 to 15.40 m, with an average of 7.60 m and a standard deviation of 2.11 m. The range of tree DBH is 2.80 to 28.65 cm, with an average of 11.38 cm and a standard deviation of 3.57 cm. The east–west crown width ranges from 1.60 to 6.60 m, with an average of 4.50 m and a standard deviation of 1.18 m. The north–south crown width ranges from 1.70 to 6.20 m, with an average of 4.26 m and a standard deviation of 0.73 m. The LAI measured using LAISmart ranges from 0.80 to 3.10, with an average of 2.02 and a standard deviation of 0.67. The measured canopy closure in the field ranges from 32.01% to 85.69%, with an average of 59.20% and a standard deviation of 13.46%. The unit area forest biomass in broadleaf forests ranges from 3.69 to 184.78 t/ha, with an average of 73.81 t/ha and a standard deviation of 42.85 t/ha.

For mixed forests in the study area, the tree height ranges from 10 to 25 m, with an average of 13.04 m and a standard deviation of 2.20 m. The range of tree DBH is 5.09 to 25.46 cm, with an average of 12.57 cm and a standard deviation of 3.44 cm. The east–west crown width ranges from 2.76 to 9.50 m, with an average of 5.14 m and a standard deviation of 1.12 m. The north–south crown width ranges from 1.30 to 8.80 m, with an average of 4.97 m and a standard deviation of 0.98 m. The LAI measured using Smart-LAI ranges from 1.10 to 3.70, with an average of 2.25 and a standard deviation of 0.54. The measured canopy closure in the field ranges from 28.15% to 82.40%, with an average of 60.25% and a standard deviation of 12.69%. The unit area forest biomass in mixed forests ranges from 3.07 to 164.71 t/ha, with an average of 71.46 t/ha and a standard deviation of 51.71 t/ha.

#### 2.2.2. Multispectral Image Data

**Figure 2:**
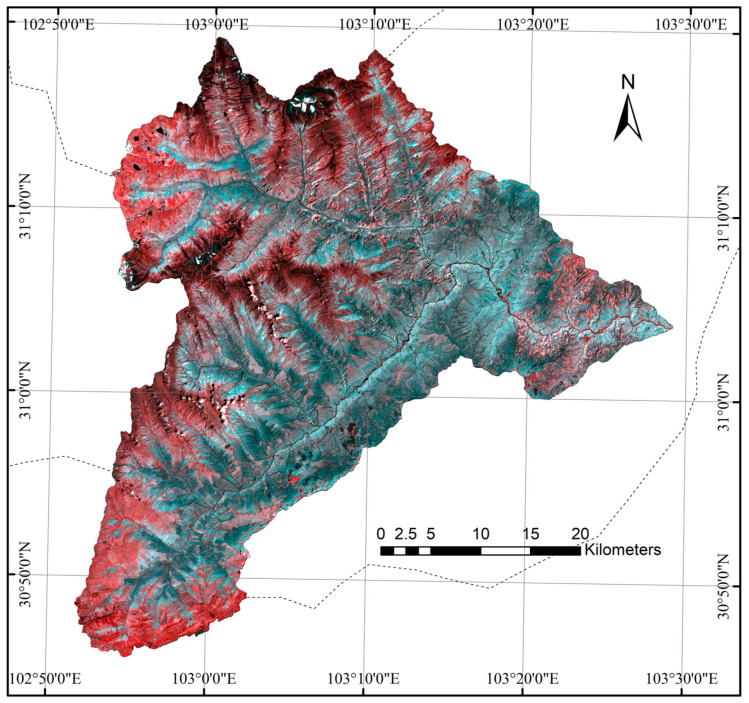
Sentinel-2 false color composite image of the Wolong Nature Reserve (14 October 2021).

The satellite multispectral data used in this study are from Sentinel-2. Sentinel-2 is a high-resolution imaging satellite equipped with a multispectral imaging sensor (MSI), operating at an altitude of 786 km and featuring 13 spectral bands with a swath width of 290 km. The ground resolution of the data includes three different levels: 10 m, 20 m, and 60 m. The revisit cycle for a single satellite is 10 days, but with two satellites in operation, the revisit cycle is reduced to 5 days. The spectral bands range from visible light and near-infrared to shortwave infrared, each with varying spatial resolutions. Among optical data sources, Sentinel-2 is unique in offering three bands within the red-edge range, making it highly effective for monitoring vegetation health. Due to cloud cover greater than 90% in the region’s imagery from September to October 2020, we selected two Sentinel-2A images from 14 October 2021 (Figure 2). The spectral bands and spatial resolution of Sentinel-2A are listed in Table 3. The visible spectral bands (B2, B3, B4) with a 10 m spatial resolution were employed in this study. The selection criteria for the imagery were as follows: (1) the images were taken during a similar vegetation growth period as the field surveys, and (2) the cloud cover was less than 30%. The data were sourced from the Google Earth Engine (GEE) platform, specifically the Sentinel-2 L2A product. This product has undergone pre-processing in GEE, including radiometric calibration and atmospheric correction. The data provided are bottom-of-atmosphere corrected reflectance values. After cloud and snow removal, the data were processed to obtain true surface reflectance values, and the area of interest was extracted and clipped within GEE.

#### 2.2.3. Terrain Data

The DEM data used in this study is sourced from the ALOS satellite data (https://urs.earthdata.nasa.gov, accessed on 1 October 2020). ALOS is the Advanced Land Observing Satellite-1 (ALOS) project by the Japan Aerospace Exploration Agency (JAXA). The data is derived from the ALOS satellite’s phased array L-band synthetic aperture radar (PALSAR). The AW3D30 version for land areas has a horizontal resolution of 12.5 m and a vertical accuracy of 2m, making it the highest resolution DEM data currently available. The ALOS-12.5 m DEM data is acquired using the Panchromatic Remote-sensing Instrument for Stereo Mapping (PRISM) sensor on the ALOS satellite. This sensor employs a three-line array camera, which can capture high-resolution stereo image data. Using these stereo images, a digital elevation model (DEM) can be generated.

**Figure 3:**
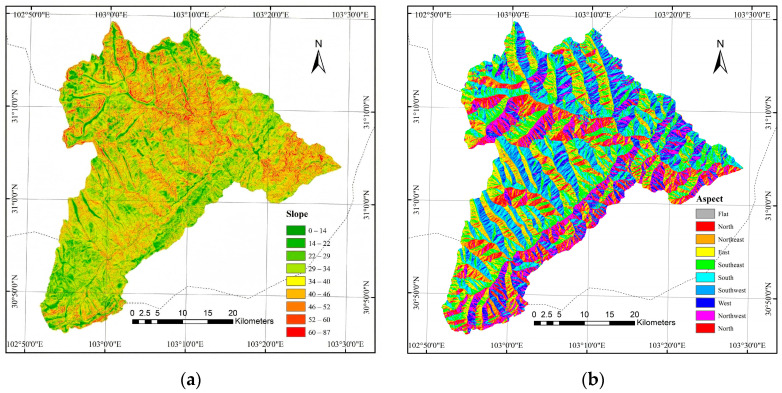
Slope (**a**) and aspect (**b**) of the Wolong Nature Reserve.

In ArcGIS 10.8, terrain indices derived from the DEM data were created, with a resolution of 10 m. Figure 3 presents the slope map and aspect map of the study area. The average slope of the study area is 31.4°, and the aspect ranges from 0° to 360°.

#### 2.2.4. Vegetation Index Extraction

Vegetation index extraction from remote sensing images is essential for improving model performance due to its strong correlation with LAI. In this study, five VIs were selected: the Normalized Difference Vegetation Index (NDVI), the Index-based Built-up Index (IBI), the Difference Vegetation Index (DVI), the Ratio Vegetation Index (RVI), and the Green Chlorophyll Index (CIg).

The Normalized Difference Vegetation Index (NDVI) is calculated by measuring the difference between visible light and infrared light reflected by vegetation. It can reflect the background influence of factors such as soil, dead leaves, snow, wetlands, and roughness, and is somewhat related to vegetation cover. NDVI values are negative when visible light is highly reflected, typically indicating cloud- or snow-covered areas. An NDVI value of zero indicates rocky or bare soil areas where the near-infrared and red bands are roughly equal. Positive NDVI values indicate vegetation cover. As vegetation coverage increases in areas with low to moderate cover, the NDVI value increases. However, once a certain threshold of coverage is reached, the increase in NDVI becomes more gradual. NDVI is less sensitive to areas with high vegetation cover, making it suitable for dynamic monitoring during early and mid-stage vegetation growth. Multiple studies have shown that NDVI is closely related to biophysical parameters such as LAI, NPP, and FAPAR [45]. The formula for NDVI is presented in Equation (5), where *NIR* represents the near-infrared band and *R* represents the red band.

(5) $$NDVI=\frac{NIR-R}{NIR+R}$$

The Ratio Vegetation Index is highly correlated with chlorophyll content, leaf biomass, tree trunk biomass, and LAI, making it crucial for detecting and estimating vegetation biomass. RVI is also somewhat related to vegetation cover, which is especially useful for monitoring vegetation with high growth and coverage. However, when the vegetation cover is less than 50%, this sensitivity decreases significantly, which makes RVI advantageous for reflecting differences in vegetation cover and growth conditions. Despite this, RVI is highly affected by atmospheric interference, which reduces its sensitivity for vegetation monitoring [46]. The formula for RVI is presented in Equation 6, where *NIR* represents the near-infrared band and *R* represents the red band.

(6) $$RVI=\frac{NIR}{R}$$

The Difference Vegetation Index is more sensitive to changes in soil background and can be applied to monitoring changes in vegetation cover. When vegetation cover is between 15% and 25%, DVI changes with AGB, increasing as biomass increases. However, when vegetation cover exceeds 80%, DVI’s sensitivity to vegetation gradually decreases. Compared to RVI, DVI is more sensitive to soil, whereas RVI is more sensitive to areas with high vegetation cover. Therefore, DVI is more suitable for the early stages of reforestation or grassland restoration, while RVI is more appropriate for later stages or the monitoring of natural forests [47]. The formula for DVI is presented in Equation (7), where *NIR* represents the near-infrared band and *R* represents the red band.

(7) DVI=NIR−R

The Index-based Built-up Index is derived by incorporating the Soil-Adjusted Vegetation Index (SAVI) and the Modified Red Edge Normalized Difference Water Index (MNDWI), which represent vegetation and water bodies. After normalization, this yields a new built-up index. The NDBI, often used for extracting built-up and bare soil areas, is affected by vegetation and water bodies, which can interfere with the accuracy of extraction. Therefore, further processing of NDBI is required [48]. There are two formulas for calculating IBI, and the one with better performance among the two was adopted in this study. The formula for IBI is presented in Equation (8), where *SWIR* represents the shortwave infrared band; *NIR* represents the near-infrared band; *R* represents the red band; *G* represents the green band.

(8) $$IBI=\frac{2SWIR}{SWIR+NIR}-\left(\frac{NIR}{NIR+R}+\frac{G}{G+SWIR}\right)/\frac{2SWIR}{SWIR+NIR}+(\frac{NIR}{NIR+R}+\frac{G}{G+SWIR})$$

The Green Chlorophyll Index is used to assess chlorophyll content in leaves and to measure pigments related to stress in vegetation. It is widely applied in ecosystem research, canopy stress analysis, and crop monitoring [49]. The formula for CIg is presented in Equation (9), where *NIR* represents the near-infrared band and *G* represents the green band.

(9) $$CIg=\frac{NIR}{G}-1$$

#### 2.2.5. Canopy Height and Leaf Area Index Extraction

**Figure 4:**
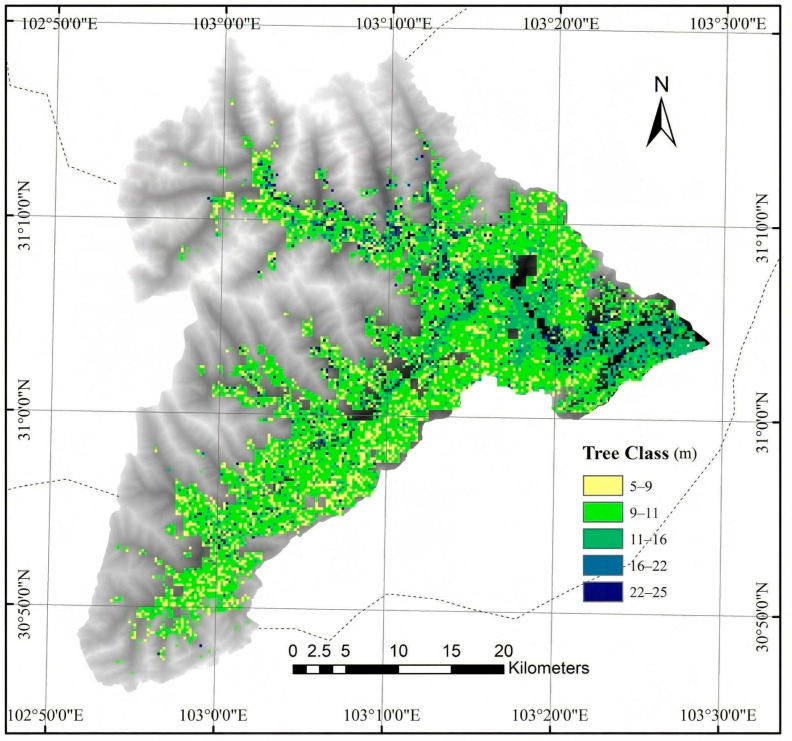
Canopy height of the Wolong Nature Reserve [40].

The canopy height data for the study area was generated using the model proposed by Liu et al. [40]. This model was selected due to its integration of InSAR data and UAV-derived data, combined with machine learning algorithms to construct a highly accurate canopy height model. Figure 4 presents the canopy height data for the study area.

**Figure 5:**
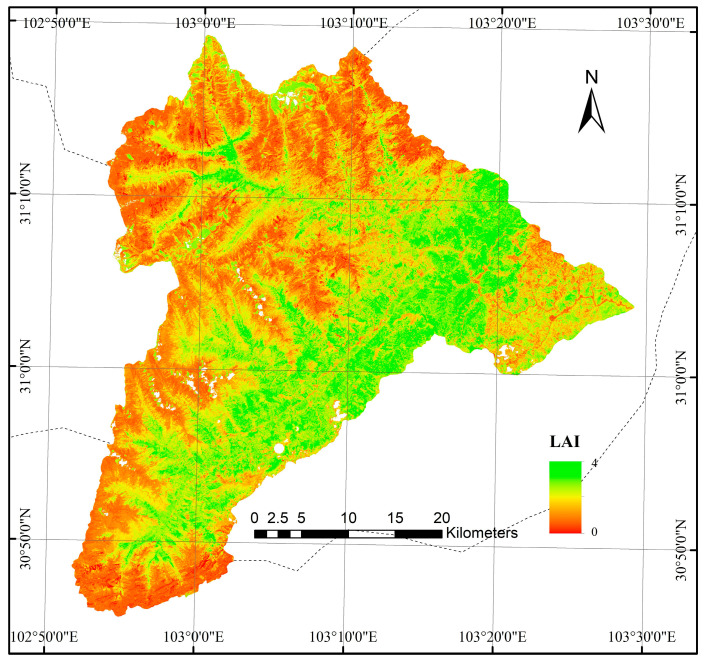
LAI of the Wolong Nature Reserve [39].

The extraction of LAI is based on the LAI estimation model proposed by Liu et al. [39]. This method demonstrates high accuracy (R^2^ = 0.83) and has been validated using field-measured data (R^2^ = 0.78). The regional estimation accuracy is higher than the field validation result of the GLASS LAI product (R^2^ = 0.74). Figure 5 presents the LAI data for the study area.

### 2.3. Correlation Analysis Between Forest Parameters and Measured AGB

**Figure 6:**
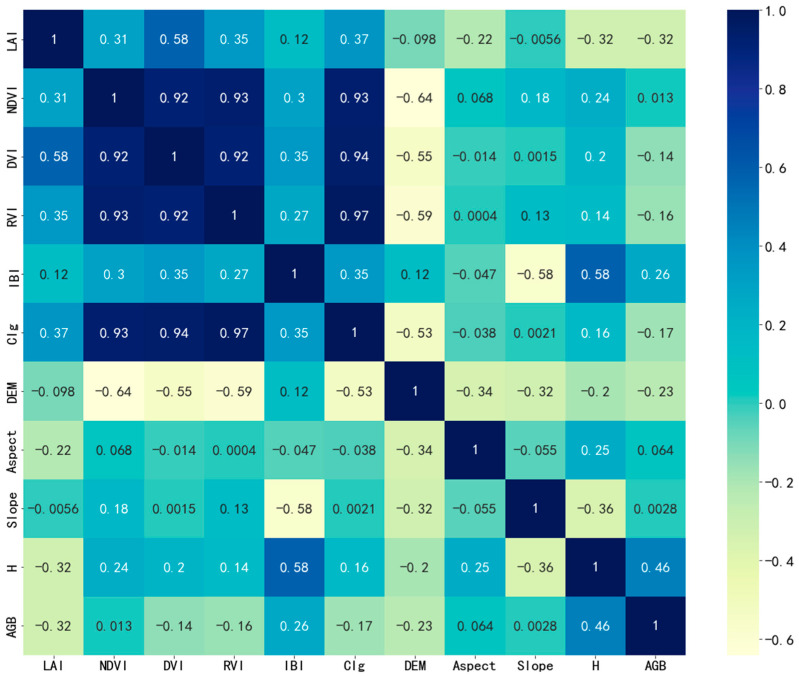
Pearson correlation analysis heatmap between measured AGB and forest parameters. The forest parameters are categorized as follows: vegetation indices: NDVI, DVI, RVI, IBI, CIg; canopy structure parameters: LAI, H; topographic parameters: DEM, aspect, slope. Significance levels were tested at the 0.05 level.

The correlation analysis was conducted between vegetation indices, topographic indices (slope and aspect), canopy height, LAI, and the measured AGB (Figure 6). The Pearson correlation coefficient was used to quantify the relationships between variables, and the correlation heatmap was generated using the seaborn library in Python (v3.9). Significance levels were tested at the 0.05 level. The measured AGB used in the analysis represents the total AGB within a 10 × 10 m plot, rather than the biomass per unit area.

The highest correlation with measured AGB was observed for tree height, with a correlation coefficient of 0.46. The leaf area index showed a weaker correlation with AGB compared to tree height but was still significantly higher than other variables. Among the topographic factors, slope and aspect had minimal contributions to AGB, both with correlation coefficients less than 0.1. Elevation exhibited a negative correlation with AGB, with a coefficient of 0.23, indicating that forest areas at higher altitudes tend to have lower biomass values.

Among all vegetation indices, IBI showed the highest contribution, with a correlation coefficient of 0.26. The correlations for the other vegetation indices ranged between 0 and 0.2.

Additionally, the correlation heatmap revealed a clear relationship between LAI and all vegetation indices, while elevation showed a significant negative correlation with all vegetation indices, indicating that vegetation distribution is influenced by ground elevation. Although AGB showed a weak correlation with slope, both tree height and LAI were correlated with slope and aspect to varying degrees.

Based on the correlation analysis results, forest AGB models were established using univariate function, multivariate regression, and decision tree algorithms. Since the main dominant tree species in the study area are large tree species such as larch and fir, forest AGB modeling was conducted for 20 coniferous forests and 29 mixed forests. Among them, data from 15 coniferous forests and 21 mixed forest plots were used to construct the model, and the remaining 13 plots were used for result validation. The data used for model development had a relatively uniform spatial distribution within the study area. However, the decision tree algorithm was not validated using these 13 sample points. We trained the model with as much data as possible; thus, 41 samples (85% of the data) were used for model training and hyperparameter tuning, and the remaining eight samples were used for result validation. Leave-One-Out Cross Validation (LOOCV) was adopted during the modeling process to ensure model accuracy.

### 2.4. Modeling

#### 2.4.1. The Forest AGB Model Based on Univariate Function

According to the correlation analysis results, tree height, which has the highest correlation with AGB, was selected to construct univariate AGB models, including linear, exponential, logarithmic, power function, and polynomial models.

#### 2.4.2. The Forest AGB Model Based on Multivariate Regression

Based on the results of the correlation analysis, multivariate regression models were established using vegetation indices, terrain indices, LAI, and the field-measured AGB of the forest. In the modeling process, the backward elimination method was used for selecting independent variables. In this method, all variables were initially included in the model, with the total number of variables being n. The significance results for each independent variable were calculated and ranked, and the variable with the largest *p*-value (greater than 0.1) was first removed from the model. Then, a regression model was calculated with the remaining n-1 variables, and the same procedure was followed to remove the variable with the lowest correlation, repeating the process until all the remaining independent variables in the model were statistically significant. This iterative process was performed multiple times, and the experimental results were calculated and compared, ensuring that all the remaining independent variables in the model had statistical significance.

In this experiment, eight regression models were established. Before modeling, all data were subjected to feature scaling using the mean normalization method. The normalization formula used is shown in Equation (10):

(10) $$x’=\frac{x-average(x)}{max(x)-min(x)}$$

In Equation (10), *x*′ represents the normalized value of the variable, *x* is the original value of the variable, *average*(*x*) denotes the mean value of the variable across all samples, and *max*(*x*) and *min*(*x*) represent the maximum and minimum values of the variable in the dataset, respectively.

The condition index is used to test for multicollinearity issues within the models. A condition index of less than 30 indicates that the variables selected in the model do not exhibit multicollinearity. A condition index between 30 and 100 indicates the presence of some degree of collinearity, while a condition index greater than 100 suggests severe multicollinearity among the variables selected for the model [50].

#### 2.4.3. The Forest AGB Model Based on Machine Learning Algorithm

A machine learning model was established using canopy height, leaf area index, vegetation indices, and terrain indices in relation to the measured AGB. Due to the limited sample size, the Classification and Regression Tree (CART) as the single decision tree algorithm, which is suitable for handling small sample data, was chosen for modeling, with the splitting criterion set as the mean squared error (MSE) to fit the continuous AGB values.

The Recursive Feature Elimination (RFE) method was used to select the optimal feature combination. RFE is a robust, model-based backward selection technique that uses feature importance metrics to iteratively remove irrelevant features. During selection, nine-fold cross-validation (CV) was employed to evaluate the performance of each feature subset, and the optimal combination of five variables was finally identified.

After determining the feature variables, GridSearch was used to find the optimal parameter values. The GridSearch algorithm is a method for optimizing model performance by exhaustively searching through combinations of given parameters. It instantiates the given model, performs CV-fold cross-validation, and selects the hyperparameter combination with the highest average score as the best choice for the returned model object. This process is applied to each combination in the hyperparameter combination list. In this experiment, the CV value was set to five, and the optimal parameter values were found: the maximum depth of the decision tree was set to five, and a node must contain at least five training samples to allow branching.

The sample data used for model training (Table 4) included the following input variables: NDVI, DVI, RVI, IBI, CIg, DEM, slope, aspect, canopy height, and LAI. The output variable was the forest’s measured AGB data.

Based on the results of the RBF model parameters and feature selection, the optimal feature combination obtained includes five feature variables: canopy height, elevation, slope, aspect, and green light chlorophyll index. Using these selected features, the model estimates the total AGB values, which includes stem biomass, branch biomass, and leaf biomass.

## 3. Results

### 3.1. Validation Results of the Univariate Function AGB Model

**Figure 7:**
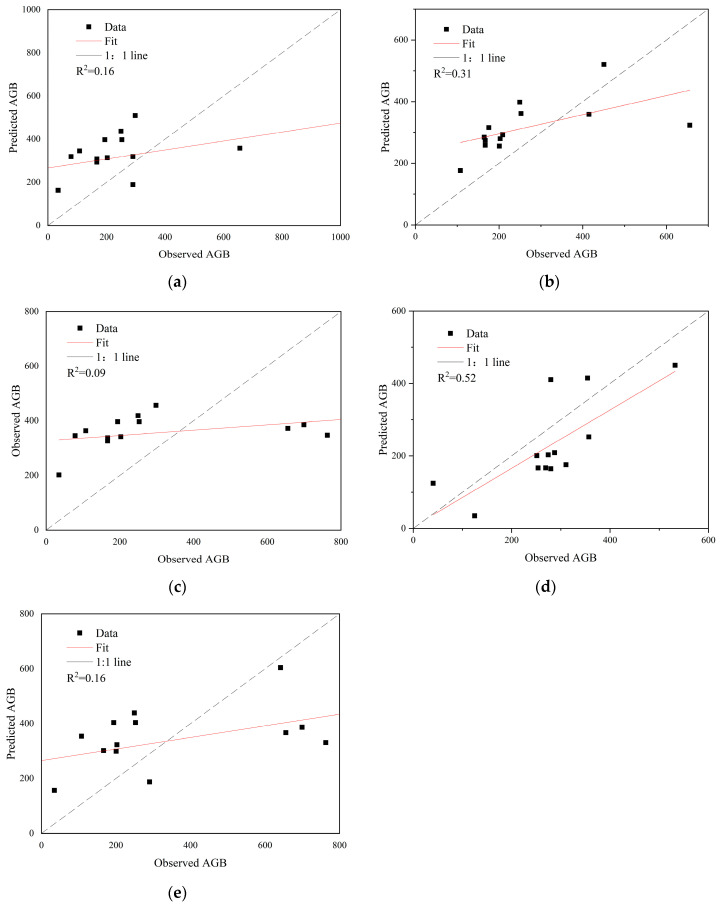
Accuracy validation of the univariate AGB model: (**a**) Linear Model Fit Accuracy; (**b**) Exponential Model Fit Accuracy; (**c**) Logarithmic Model Fit Accuracy; (**d**) Power Function Model Fit Accuracy; (**e**) Polynomial Model Fit Accuracy.

The forest AGB model established using the univariate function is shown in Table 5. The power function model for the tree height–AGB relationship exhibits the highest model accuracy with an R^2^ = 0.7349. The relationship between tree height and AGB follows a power function distribution, while the exponential function model achieves the second-highest accuracy. The remaining sample points were used for data accuracy validation (Figure 7). The forest AGB values were calculated using the linear, exponential, logarithmic, power function, and polynomial models, and linear regression was performed by comparing the predicted AGB values with the measured values. The model fit (R^2^) for each model was as follows: 0.16 for the linear model, 0.31 for the exponential model, 0.09 for the logarithmic model, 0.52 for the power function model, and 0.16 for the polynomial model.

In comparison with the 1:1 line, it is observed that the AGB values estimated by the linear model are higher than the measured AGB values, indicating a lower accuracy with significant overestimation. The exponential model has a higher accuracy than the linear model, with its estimated values more evenly distributed near the proportional line; however, overestimation still exists. The logarithmic function model has the lowest accuracy among all models, with an R^2^ value of only 0.09. The power function model demonstrates the highest accuracy, showing a certain predictive ability. Although the relationship between tree height and AGB follows a significant power function, the power function model significantly underestimates AGB in the accuracy evaluation with the measured AGB. This underestimation could be due to the dataset used for validation. Furthermore, the polynomial function model also exhibits some overestimation in its accuracy evaluation. The results from the univariate model evaluation suggest that the tree height–AGB model constructed with the function equation has relatively low overall accuracy. Using only the single variable, tree height is insufficient for regional AGB estimation.

### 3.2. Validation Results of the Multivariate Regression AGB Model

Table 6 presents all the regression model equations established using the backward elimination method, along with their corresponding fit coefficients and root mean square error (RMSE).

Multicollinearity issues were found between parameters with linear relationships. The presence of collinearity can lead to instability in regression results, where the values of the regression coefficients change significantly when sample points or features are added or removed. The results of the multicollinearity diagnostics are shown in Table A1 (Appendix A), which lists the variable dimensions for each model, the eigenvalues of the variables, and the condition index for each variable within the model. However, during the model-building process, variables with severe multicollinearity were progressively removed.

**Figure 8:**
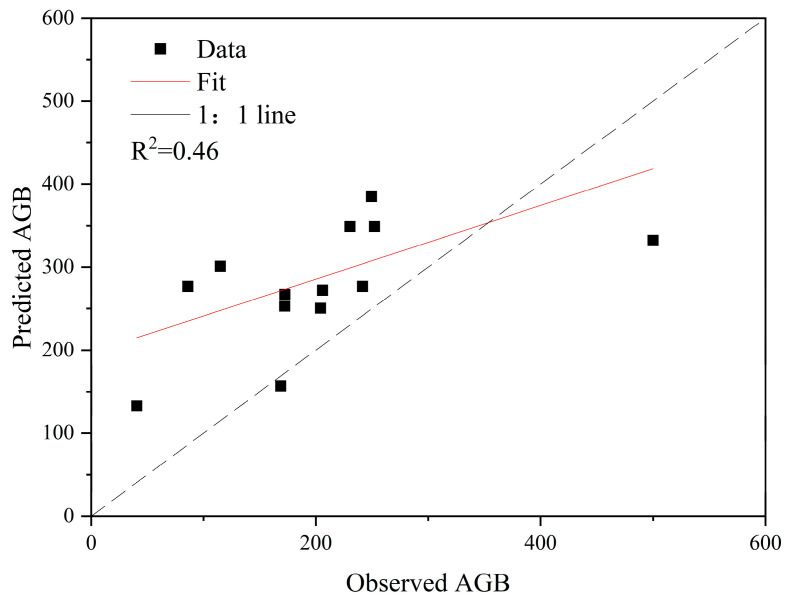
Accuracy validation of the optimal multivariate AGB model.

The model with the highest accuracy is Model 7, which includes variables such as LAI, IBI, and CIg. The model achieved an R^2^ value of 0.517, which is the highest fitting degree among the eight regression models and outperforms the previous univariate linear regression models, coming second only to the univariate power function model. Table A1 indicates that all parameters in this model have a condition index below 30 (the model ID and corresponding parameters have been bolded in Table A1 for clarification). The remaining 13 sample points were used for model validation (Figure 8), with a precision evaluation result of R^2^ = 0.46. A comparison with the 1:1 line reveals that the AGB values estimated by this model are higher than the measured AGB values, indicating a slight overestimation. Nonetheless, the accuracy of the forest AGB model based on multivariate is significantly higher than that of all other AGB models (both univariate and multivariate), except for the power function model, as discussed in the previous section.

### 3.3. Validation Results of the Machine Learning AGB Model

**Figure 9:**
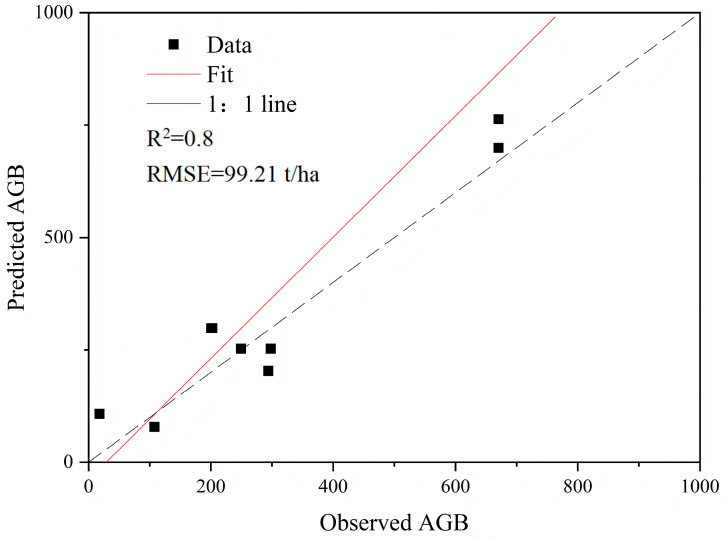
Accuracy validation of the forest AGB decision tree model.

After tuning and cross-validation, the biomass model established using CART achieved high accuracy, with an R^2^ of 0.8. The remaining eight samples were used for result validation. As seen from the accuracy results validated with field-measured data (Figure 9), there is a strong linear relationship between the measured and predicted AGB values, with both the measured and predicted values evenly distributed around the 1:1 line. The R^2^ value is 0.8, and the RMSE is 99.21 t/ha. The validation accuracy is consistent with the model accuracy. This phenomenon may be attributed to the small number of validation samples and a certain degree of randomness.

This method shows high accuracy; compared to univariate function models and multivariate regression models, the forest AGB model built using decision trees does not exhibit the overestimation of AGB values.

## 4. Discussion

This study presents a comparative analysis of forest aboveground biomass (AGB) estimation models, demonstrating that a decision tree-based machine learning model integrating canopy height, leaf area index, vegetation indices, and topographic indices outperforms traditional univariate functions and multivariate regression models. The machine learning model achieved an R^2^ of 0.8, significantly reducing the overestimation bias observed in both canopy height-based power function models (R^2^ = 0.7349) and multivariate regression models (best R^2^ = 0.517). This finding highlights the superiority of machine learning in capturing non-linear relationships and complex interactions among forest structural parameters, which is crucial for accurate AGB estimation in heterogeneous landscapes like Wolong Nature Reserve.

Traditional AGB estimation models often rely on diameter at breast height or tree height through allometric equations (e.g., power functions), which primarily represent stem biomass and overlook branch and leaf contributions. In contrast, our multi-parameter approach integrates VIs (e.g., NDVI, IBI), LAI, and canopy height, which are closely linked to photosynthetic activity and canopy structure, thereby capturing total AGB more comprehensively. The superior performance of the machine learning model compared to univariate models (such as the power function with R^2^ = 0.52 in the validation stage) confirms that incorporating ecological parameters beyond tree height can enhance estimation accuracy.

The decision tree model’s effectiveness stems from its ability to handle non-linear relationships and variable interactions. For example, it identifies IBI and CIg as key predictors, reflecting their sensitivity to canopy chlorophyll content and urban–rural land cover transitions. Additionally, the model’s integration of LAI-a critical parameter for photosynthetic carbon assimilation addresses a common gap in traditional models that focus solely on stem biomass [15].

The spatial heterogeneity in AGB distribution underscores the importance of species-specific allometry. Coniferous forests in lower elevations exhibit higher AGB despite similar heights to mixed forests, likely due to denser wood structures and longer growth cycles of larch and spruce. This finding supports the need for species-specific modeling, as opposed to uniform allometric equations, with implications for regional carbon stock assessments under climate change.

Compared with forest AGB estimation models in relatively flat areas, our model shows significant advantages. Recent studies have indicated that when integrating multi-source remote sensing data for forest AGB estimation in forests below 3000 m with relatively flat terrain, the R^2^ values of different models range from 0.61 to 0.84 [51,52,53]. However, these models often struggle in mountainous regions due to complex topographic conditions. The Wolong Nature Reserve, characterized by an average slope of 31.4° and significant elevation variations (898–6132 m), presents unique challenges that plain forest models cannot adequately address. The inclusion of topographic parameters (elevation, slope, aspect) in our model is not merely a methodological choice but a necessity driven by the fundamental relationships between terrain characteristics and forest biomass accumulation. Fayad et al. demonstrated that terrain slope significantly affects GEDI-based forest height and wood volume estimation, with slopes > 20° introducing substantial errors in waveform interpretation and a degradation in accuracy (increased RMSE) of a maximum of 20 m^3^/ha was observed for slopes between 20 and 45% in wood volume estimation [54]. This finding underscores the importance of incorporating slope and aspect as direct predictors in AGB models rather than relying solely on post-processing corrections.

In addition, optical sensors are prone to saturation in dense forest areas with canopy closure greater than 80%: vegetation indices such as NDVI tend to reach asymptotic values and cannot distinguish differences in AGB. This model alleviates this problem from two aspects: First, it uses less saturation-prone vegetation indices (e.g., IBI, CIg), which remain responsive to biomass variations in highly covered areas. Second, it integrates optical data with canopy height derived from the fusion of InSAR and UAV data, which directly reflects the vertical structure and is not affected by optical saturation. This multi-approach combination is superior to single-index correction methods. Gitelson et al. confirmed that CIg mitigates saturation in dense vegetation better than NDVI [49], and this model further enhances this advantage by combining CIg, LAI, and canopy height. Although canopy height data derived from active microwave sensors was used in this study to alleviate part of the spectral saturation problem, there is still no universal method available for improving biomass estimation in densely vegetated areas despite the overall advancement in sensor performance and algorithm development. Further research is required to understand the intrinsic relationship between spectral reflectance observations and vegetation type, leaf orientation, as well as vertical and horizontal structural parameters in dense vegetation regions [55].

In addition, this study has several limitations: (1) As the study area is a typical mountain ecosystem with steep terrain and a wide elevation range, the field-collected sample was constrained, resulting in insufficient sampling of high-altitude regions (above 4200m) and rare species. (2) The model did not consider soil nutrients or climatic variables. The estimated biomass represents aboveground biomass in the region, while belowground biomass also influences regional biomass accumulation.

Future research directions may involve integrating soil carbon and nutrient datasets to refine mechanistic models; incorporating LiDAR data or hyperspectral remote sensing data to improve canopy structure characterization; validating the model using multi-year independent field surveys to assess temporal stability; and expanding the application scope of the model by integrating canopy height products (e.g., Liu X et al. [56]) to verify its applicability in other regions.

## 5. Conclusions

This study established univariate function models (based on tree height), multivariate regression models (incorporating LAI, vegetation indices, and topographic parameters), and machine learning models for forest aboveground biomass estimation. The experimental results are as follows:

1. Univariate models based on tree height

Five functional models were developed using tree height and measured AGB. Results showed that the relationship between tree height and AGB could be approximated by a power function. However, validation with remaining samples revealed significant underestimation in the power function model, while other models exhibited varying degrees of overestimation. Although the power function model demonstrated some predictive capability, relying solely on a single variable (tree height) to establish relationships with AGB failed to achieve accurate forest AGB estimation, with overall low model precision.

2. Multivariate regression models based on vegetation indices, LAI, and topographic parameters

Using a backward elimination parameter input method, eight regression models were constructed. After collinearity diagnosis, the highest-precision model was selected. Compared with the univariate functional model, this model showed a significant improvement in accuracy and exhibited certain predictive ability. However, it still failed to estimate regional-scale AGB accurately, also suffering from overestimation.

3. Decision tree model based on 10 features (vegetation indices, topographic parameters, canopy height, LAI, etc.)

The model underwent parameter tuning and was validated using cross-validation. Results indicated that the AGB model established by the decision tree algorithm outperformed univariate function models and multivariate regression models, with significantly higher precision. It showed the highest accuracy among the three methods and was applicable for estimating forest AGB in the study area.

By comparing three different methods, a total of 14 AGB models was constructed. The optimal AGB model, established via machine learning algorithms, differs from traditional forest AGB estimation methods in that it considers not only the relationships between forest parameters and stem biomass but also those between forest parameters and branch/leaf biomass, enabling more comprehensive estimation of forest aboveground biomass. The AGB obtained from this optimal model is closer to the true total AGB values.

Under the national carbon neutrality goal, accurate estimation of forest parameters (e.g., forest AGB) to analyze the carbon sequestration potential of forest ecosystems and evaluate the spatial distribution mechanism of forest carbon storage is of great scientific significance for achieving carbon neutrality and regional sustainable development. The establishment of the forest AGB model for Wolong Nature Reserve also provides scientific support for ecological protection strategies in this reserve.

## Figures and Tables

**Table 1:** Growth equation coefficients for stem, branch, and leaf AGB of dominant tree species in the study area.

Tree Species	Tree Component	*a*	*b*
Larch (*Larix gmelinii*)	Stem	0.025	0.96
	Branches	0.0021	0.9638
	Leaves	0.00126	0.9675
Spruce (*Picea* spp.)	Stem	0.0567	2.4753
	Branches	0.0116	2.4054
	Leaves	0.0083	2.3733
Fir (*Abies* spp.)	Stem	0.0238	0.9363
	Branches	0.0005	0.9105
	Leaves	0.0036	0.8974

**Table 2:** Summary statistics (minimum, maximum, mean, and standard deviation) of tree height, diameter at breast height, crown dimensions, leaf area index, canopy closure, and aboveground biomass across coniferous, broadleaf, and mixed forests in the 64 sample plots.

Forest Type		H (m)	DBH (cm)	EW-d (m)	NS-d (m)	LAI	CC (%)	AGB (t/ha)
Coniferous Forest CF	Min	8.90	15.00	3.70	2.10	1.80	20.56	1.17
	Max	22.20	27.53	7.60	8.80	2.70	95.89	194.23
	Mean	15.50	15.09	5.70	5.33	2.33	61.23	86.23
	SD	2.23	3.30	0.91	0.85	0.56	13.75	35.74
Broadleaf Forest BF	Min	2.30	2.80	1.60	1.70	0.80	32.01	3.69
	Max	15.40	28.65	6.60	6.20	3.10	85.69	184.78
	Mean	7.60	11.38	4.50	4.26	2.02	59.20	73.81
	SD	2.11	3.57	1.18	0.73	0.67	13.46	42.85
Mixed Forest MF	Min	10.00	5.09	2.76	1.30	1.10	28.15	3.07
	Max	25.00	25.46	9.50	8.80	3.70	82.40	164.71
	Mean	13.04	12.57	5.14	4.97	2.25	60.25	71.46
	SD	2.20	3.44	1.12	0.98	0.54	12.69	51.71

Abbreviations: H: tree height (m); DBH: diameter at breast height (cm); EW-d: east–west crown width (m); NS-d: north–south crown width (m); LAI: leaf area index; CC: canopy closure (%); AGB: aboveground biomass (t/ha).

**Table 3:** Sentinel-2 band data.

Sentinel-2 Bands	Wavelength (nm)	Resolution (m)
Band1-Coastal aerosol	433–453	60
Band2-Blue	458–523	10
Band3-Green	543–578	10
Band4-Red	650–680	10
Band5-Red Edge	698–713	20
Band6-Red Edge	733–748	20
Band7-Red Edge	773–793	20
Band8-NIR	785–900	10
Band9-Water vapor	935–955	60
Band10-SWIR-Cirrus	1360–1390	60
Band11-SWIR-1	1565–1655	20
Band12-SWIR-2	2100–2280	20

**Table 4:** Decision tree training sample data (partial).

ID	NDVI	DVI	RVI	IBI	CIg	DEM	Slope	Aspect	H	LAI	AGB
1	0.72	0.18	6.35	0.06	2.22	2051	32.06	308.52	10.87	1.56	205.93
2	0.78	0.28	8.38	0.09	3.43	1584	29.40	62.53	11.46	1.82	230.27
3	0.46	0.13	8.45	0.09	1.28	1790	20.95	139.24	11.96	1.69	233.02
4	0.78	0.31	2.75	0.06	3.37	2111	17.63	114.15	8.52	1.55	209.48
5	0.29	0.07	1.81	0.07	0.84	3407	17.86	65.85	8.15	1.45	168.64
6	0.66	0.21	4.99	0.05	1.48	1998	23.63	294.23	14.18	1.9	293.95
7	0.75	0.22	7.02	0.1	2.87	1971	15.04	315	12.15	1.14	257.23
8	0.52	0.08	3.25	0.03	0.54	2472	45.34	348.02	21.68	1.8	221.31

**Table 5:** Univariate function models for forest AGB.

ID	Model Type	Function	R^2^
1	Linear Model	AGB=26.038H−71.558	0.2079
2	Exponential Model	$AGB=7.206e^{0.2102H}$	0.5179
3	Logarithmic Model	AGB=280.41ln(H)−414.3	0.191
4	Power Function Model	$AGB=0.1041H^{2.813}$	0.7349
5	Polynomial Model	$AGB=-0.3818H^{2}+37.754H-151.9$	0.2118

**Table 6:** Multivariate regression models for forest AGB.

ID	Model	R^2^	RMSE
1	AGB = 1149.625 − 271.649LAI − 321.279SLOPE − 116.638ASPECT + 472.541NDVI + 87.399DVI − 96.9RVI + 489.032IBI − 163.534DEM − 1478.813CIG	0.314	182.480
2	AGB = 1161.680 − 248.940LAI − 319.337SLOPE − 179.296ASPECT + 504.663NDVI − 104.785RVI + 483.701IBI − 157.780DEM − 1453.815CIG	0.390	172.103
3	AGB = 1171.117 − 248.478LAI − 327.316SLOPE − 194.775ASPECT + 512.840NDVI + 479.735IBI − 156.904DEM − 1570.338CIGI	0.449	163.485
4	AGB = 1336.251 − 266.4LAI − 300.044SLOPE − 249.188ASPECT + 523.730NDVI + 412.815IBI − 1706.387CIG	0.490	157.293
5	AGB = 967.668 − 253.569LAI − 186.123SLOPE + 339.069NDVI + 585.902IBI − 1386.654CIG	0.482	158.594
6	AGB = 856.437 − 228.105LAI + 298.005NDVI + 583.392IBI − 1313.452CIG	0.499	155.934
7	AGB = 1045.897 − 245.174LAI + 605.571IBI − 1164.897CIG	0.517	153.064
8	AGB = 914.986 + 604.650IBI − 1240.770CIG	0.454	162.799

## Data Availability

Part of the data is available from the corresponding author upon reasonable request.

## Abbreviations

The following abbreviations are used in this manuscript:

AGB	Aboveground biomass
LAI	Leaf area index
VIs	Vegetation indices
DBH	Diameter at breast height
GEE	Google Earth Engine
RMSE	Root-mean-square error

## Appendix A

**Table A1:** Model parameter collinearity diagnosis.

ID	Dimension	Eigenvalue	Condition Index
	1	8.735	1.000
	2	1.377	2.519
	3	0.435	4.480
	4	0.215	6.374
	5	0.121	8.493
1	6	0.056	12.476
	7	0.032	16.609
	8	0.018	21.931
	9	0.005	40.188
	10	0.003	51.200
	11	0.002	67.278
	1	7.888	1.000
	2	1.251	2.511
	3	0.424	4.313
	4	0.213	6.083
2	5	0.114	8.310
	6	0.056	11.861
	7	0.032	15.800
	8	0.014	23.442
	9	0.005	39.005
	10	0.002	61.825
	1	7.222	1.000
	2	0.953	2.753
	3	0.415	4.173
	4	0.209	5.876
3	5	0.110	8.118
	6	0.049	12.158
	7	0.032	15.123
	8	0.009	27.787
	9	0.002	58.085
	1	6.579	1.000
	2	0.704	3.058
	3	0.400	4.057
	4	0.169	6.243
4	5	0.093	8.429
	6	0.043	12.386
	7	0.010	26.229
	8	0.004	41.906
	1	5.937	1.000
	2	0.703	2.905
	3	0.170	5.906
5	4	0.110	7.349
	5	0.056	10.253
	6	0.017	18.439
	7	0.005	33.218
	1	5.095	1.000
	2	0.689	2.719
6	3	0.121	6.496
	4	0.068	8.636
	5	0.021	15.610
	6	0.006	29.996
	1	4.244	**1.000**
	2	0.566	**2.737**
**7**	3	0.118	**6.001**
	4	0.059	**8.462**
	5	0.013	**18.401**
	1	3.362	1.000
8	2	0.557	2.458
	3	0.069	7.002
	4	0.013	16.144

The bold text indicates the condition index of the best-performing model in this table. Values below 30 suggest no multicollinearity between model parameters.
